# Multimodal Imaging of Brain Activity to Investigate Walking and Mobility Decline in Older Adults (Mind in Motion Study): Hypothesis, Theory, and Methods

**DOI:** 10.3389/fnagi.2019.00358

**Published:** 2020-01-08

**Authors:** David J. Clark, Todd M. Manini, Daniel P. Ferris, Chris J. Hass, Babette A. Brumback, Yenisel Cruz-Almeida, Marco Pahor, Patricia A. Reuter-Lorenz, Rachael D. Seidler

**Affiliations:** ^1^Department of Aging and Geriatric Research, University of Florida, Gainesville, FL, United States; ^2^Brain Rehabilitation Research Center, Malcom Randall VA Medical Center, Gainesville, FL, United States; ^3^Department of Biomedical Engineering, University of Florida, Gainesville, FL, United States; ^4^Department of Applied Physiology and Kinesiology, University of Florida, Gainesville, FL, United States; ^5^Department of Biostatistics, University of Florida, Gainesville, FL, United States; ^6^Pain Research and Intervention Center of Excellence, University of Florida, Gainesville, FL, United States; ^7^Department of Psychology, University of Michigan, Ann Arbor, MI, United States

**Keywords:** mobility, walking, older adults, brain, neuroimaging, EEG, MRI, fNIRS

## Abstract

Age-related brain changes likely contribute to mobility impairments, but the specific mechanisms are poorly understood. Current brain measurement approaches (e.g., functional magnetic resonance imaging (fMRI), functional near infrared spectroscopy (fNIRS), PET) are limited by inability to measure activity from the whole brain during walking. The *Mind in Motion Study* will use cutting edge, mobile, high-density electroencephalography (EEG). This approach relies upon innovative hardware and software to deliver three-dimensional localization of active cortical and subcortical regions with good spatial and temporal resolution during walking. Our overarching objective is to determine age-related changes in the central neural control of walking and correlate these findings with a comprehensive set of mobility outcomes (clinic-based, complex walking, and community mobility measures). Our hypothesis is that age-related walking deficits are explained in part by the Compensation Related Utilization of Neural Circuits Hypothesis (CRUNCH). CRUNCH is a well-supported model that describes the over-recruitment of brain regions exhibited by older adults in comparison to young adults, even at low levels of task complexity. CRUNCH also describes the limited brain reserve resources available with aging. These factors cause older adults to quickly reach a ceiling in brain resources when performing tasks of increasing complexity, leading to poor performance. Two hundred older adults and twenty young adults will undergo extensive baseline neuroimaging and walking assessments. Older adults will subsequently be followed for up to 3 years. Aim 1 will evaluate whether brain activity during actual walking explains mobility decline. Cross sectional and longitudinal designs will be used to study whether poorer walking performance and steeper trajectories of decline are associated with CRUNCH indices. Aim 2 is to harmonize high-density EEG during walking with fNIRS (during actual and imagined walking) and fMRI (during imagined walking). This will allow integration of CRUNCH-related hallmarks of brain activity across neuroimaging modalities, which is expected to lead to more widespread application of study findings. Aim 3 will study central and peripheral mechanisms (e.g., cerebral blood flow, brain regional volumes, and connectivity, sensory function) to explain differences in CRUNCH indices during walking. Research performed in the *Mind in Motion Study* will comprehensively characterize the aging brain during walking for developing new intervention targets.

## Introduction

Mobility disability impacts approximately 30% of individuals aged 60–69, 40% of individuals aged 70–79, and 50% of individuals age 80 or older (Seeman et al., 2010), resulting in more than $42 billion in health care costs (Hardy et al., 2011). Preserving walking ability with advancing age is central to maintaining a high quality of life, including retention of many activities necessary for full independence in the community. Research is needed to identify and target neural mechanisms in order to reduce mobility disability. We are therefore conducting the Mind in Motion Study, funded by the National Institute on Aging (RFA-AG-18-019; U01AG061389), to assess brain control of walking in the context of age-related decline of mobility function. This study will advance knowledge about the mechanisms by which brain aging contributes to functional decline, in order to guide the development of future interventions.

**FIGURE 1:**
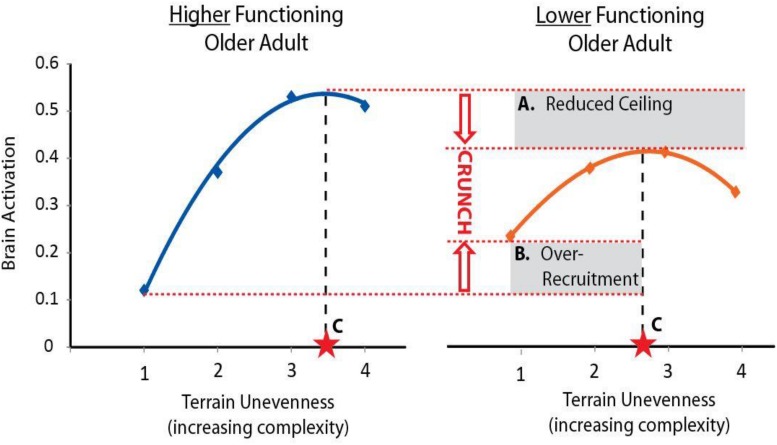
Conceptual figure of CRUNCH. The *Compensation-Related Utilization of Neural Circuits Hypothesis (CRUNCH)* is an evidence-based framework for interpreting brain activity during tasks of increasing complexity. Here we show a conceptual figure of brain activity (arbitrary units) versus levels of task complexity (terrain unevenness). CRUNCH in older adults with lower function **(right)** is characterized by: **(A)** reduction in the brain resource ceiling, and **(B)** over-recruitment of brain resources at lower levels of task difficulty. Brain activity plateaus or decreases when the ceiling is reached, and task performance suffers. For use in statistical models, the CRUNCH concept can be summarized as a single value, **(C)** task complexity at the inflection where brain activity plateaus or begins to decline.

A challenging aspect of studying brain control of task performance is that both higher and lower levels of brain activity might convey a benefit depending on the context of the task and individual. To guide interpretation of walking-related brain activity we will use the theoretical framework provided by the Compensation Related Utilization of Neural Circuits Hypothesis (CRUNCH) (Reuter-Lorenz and Cappell, 2008). CRUNCH describes two major age-related changes in brain network recruitment (Figure 1). The first is compensatory over-recruitment of brain networks, even at low levels of task difficulty. In the context of walking, over-recruitment may be elicited by factors within and peripheral to the brain (Clark, 2015). Factors within the brain include inefficient processing and poor specificity of network recruitment. Both lead to greater and/or more widespread brain activity to achieve task performance (Cabeza et al., 2002; Reuter-Lorenz and Cappell, 2008). Factors peripheral to the brain may include control impairments at other levels of the neuraxis (e.g., brainstem, cerebellar, or spinal networks) or impaired input from sensory systems including vision, somatosensation, and proprioception (Nielsen, 2003; Clark, 2015). The demand for brain resources to control walking may increase to compensate for these deficits. Regardless of the specific cause(s) of over-recruitment, this phenomenon encumbers resources and exacerbates the consequences of the second detrimental CRUNCH effect, which is reduced availability of brain resources (Reuter-Lorenz and Cappell, 2008). Although compensatory over-recruitment may help to prevent declines in performance at lower levels of task complexity, the cumulative effect of CRUNCH is that the ceiling in brain resources is reached quickly (e.g., at moderate levels of task complexity). As task complexity increases further, the demand for brain resources exceeds the ceiling, and task performance suffers (Reuter-Lorenz and Cappell, 2008).

The overarching hypothesis for the Mind in Motion Study is that CRUNCH-related indices of brain activity during walking will be associated with baseline and prospectively measured mobility outcomes in older adults. The primary objective is to determine the extent to which brain over-recruitment and ceiling effects, particularly in frontoparietal and anterior cingulate regions, relate to poor mobility. Our primary measure of brain activity during walking is an innovative approach using high-density electroencephalography (EEG). This approach delivers three-dimensional localization of active cortical and subcortical brain regions with high spatial and temporal resolution. Cortical activity during walking will also be assessed with functional near infrared spectroscopy (fNIRS). A second objective is to characterize and understand the consistency of brain activity data across three modalities (EEG, fNIRS, fMRI) during actual and imagined walking. We refer to this as the harmonization aim. A third objective is to investigate mechanisms contributing to compensatory brain over-recruitment (e.g., peripheral sensorimotor impairments) and ceiling effects (e.g., brain structure and perfusion) during walking.

## Study Procedures

### Participants

The Mind in Motion study enrollment criteria are intended to yield a generalizable sample of older adults, including people at risk of future mobility disability. The criteria also are designed to avoid health factors that would significantly interfere with walking ability yet are not directly related to brain function. We seek to enroll 200 older adults and 20 young adults. Enrollment criteria are listed in Table 1. All young adult participants will be healthy and high functioning. For the older adult participants, we will enroll people with a broad range of mobility function based on Short Physical Performance Battery (SPPB) score (Guralnik et al., 1994). A small proportion of older adults will be high functioning with SPPB = 10 (out of 12 possible points). The majority will have scores less than 10. All participants must be capable of completing a 400 m walk test at baseline.

**TABLE 1:** Enrollment criteria.

**Inclusion criteria**
• Community dwelling men and women 70 (years old; men and women aged 20–40 years old • Able to complete the 400 m walk test within 15 min without sitting or the help of another person or a walker • Willingness to undergo all testing procedures • English speaking • Willingness to be enrolled for 1.25–3 years, depending on enrollment date **Exclusion criteria**
• Significant medical event requiring hospitalization in the past 6 months • Severe visual impairment or corrected visual acuity less than 20/40 Not meeting MRI eligibility • Clinically diagnosed vestibular dysfunction • Unwilling or unable to do an over-ground version of the uneven terrain task without assistive device • Develops chest pain or severe shortness of breath during physical stress • History of stroke • History of clinically diagnosed traumatic brain injury • Diagnosis of dementia or taking cholinesterase inhibitors • Any major ADL disability (unable to feed, dress, bath, use the toilet, or transfer) • Report of lower extremity pain due to osteoarthritis that significantly limits mobility • Diagnosis or treatment for rheumatoid arthritis • Lives in a nursing home (assisted living will not be excluded) • Receiving physical therapy for gait, balance, or other lower extremity condition • Known neuromuscular disorder or overt neurological disease • Unable to communicate because of severe hearing loss or speech disorder • Planned surgical procedure or hospitalization in the next 12 months • Severe pulmonary disease, requiring the use of supplemental oxygen • Terminal illness, as determined by a physician • Known cardiac disease • Planning to move out of the area in next year, or leave the area for >6 months during follow-up • Other significant conditions discovered during medical screening that would impact safety and/or compliance • Use of walker or wheel chair • Failure to provide informed consent • Transaminases greater than twice upper limit of normal • Hemoglobin < 10 g/dL **Temporary exclusion criteria** • Clinically significant abnormalities in blood chemistry • Severe hypertension (e.g., systolic > 200; diastolic > 110 mmHg) • Uncontrolled diabetes or hyperglycemia • Other temporary intervening events, such as sick spouse, bereavement, or recent move • Other conditions identified with medical history at enrollment that places the participant at risk for participation

### Study Design

The Mind in Motion Study is designed to maximize opportunities for discovering age-related mediators of brain activity during walking. Therefore, the experimental design includes nested studies, measures of both central and peripheral mechanisms, and a broad array of walking outcome measures. We will conduct a longitudinal, prospective cohort study with a follow-up of 1.2–3.5 years (dependent upon time of enrollment). At baseline, participants will undergo extensive assessment of mobility and brain function, as described below. Participants will then attend follow-up sessions at 6-month intervals to assess changes in mobility function over time. Nested within this design is a longitudinal, case-control sub-study. A “case” is a participant who exhibits major mobility disability (MMD) upon return for a follow-up visit, as defined by inability to complete the 400 m walk assessment (Pahor et al., 2014). When an MMD case is identified during longitudinal follow-up, that person will be referred for an additional session of EEG measurement during walking, in order to assess changes since baseline. A “control” participant without MMD (matched by sex, age, and approximate enrollment duration) will also be referred for comparison. An additional nested sub-study will involve 90 participants who will undergo a multi-modal neuroimaging battery at baseline, with the objective of harmonizing brain activity findings across EEG, fMRI, and fNIRS.

Preliminary screening of all participants will be conducted by telephone, followed by an onsite screening visit in older adults to assess full eligibility. Eligible participants will attend five separate study visits for baseline assessments according to the schedule in Table 2. Each participant will generally complete all of the baseline assessment visits within a 1 month period. The length of a visit may be up to 5 h, but most visits will be shorter duration. Table 2 also shows the assessments that will be performed for the follow-up visits, case/control sub-study, and for harmonization across neuroimaging techniques.

**TABLE 2:** Assessment schedule.

	**Screening and**	**6 month**	**12 month**	**18 month**	**24 month**	**30 month**	**36 month**	**42 month**	**Case**
	**baseline**								**control**
Screening visit consent	SV								
Short physical performance battery	SV	X	X	X	X	X	X	X	
Eligibility screening	SV								
Full study consent	BV #1								
Demographics	BV #1								
Medical history, medications, general health, disability questionnaires	BV #1	X	X	X	X	X	X	X	
Cognition (NIH toolbox)	BV #1	X	X	X	X	X	X	X	
Blood draw	BV #1								
400 m walk test	BV #1	X	X	X	X	X	X	X	
Instrumented gait mat	BV #1	X	X	X	X	X	X	X	
Community mobility	BV #1	X	X	X	X	X	X	X	
Sensory measures	BV #1								X
EEG uneven terrain walking	BV #2								X
EEG (imagined walking)^∗^	BV #2								
fNIRS uneven terrain walking^∗^	BV #3								
fNIRS imagined walking^∗^	BV #3								
Complex walking w/biomechanics	BV #4				X				X
MRI structural and resting functional connectivity, cerebral perfusion	BV #5								
fMRI imagined walking^∗^	BV #5								
Interim Health events/conditions		X	X	X	X	X	X	X	

*SV, screening visit; BV, baseline visit; EEG, electroencephalography; fNIRS, functional near infrared spectroscopy; fMRI, functional magnetic resonance imaging. ^∗^items marked by an asterisk are acquired for a subset of participants to address harmonization across neuroimaging modalities.*

### Participant Characteristics

All study participants will be thoroughly characterized at baseline and at each follow-up visit. Information collected will include age, sex, race, ethnicity, marital status, education, income, body mass index, alcohol use, smoking history, depression symptoms, fall history, current health status, medication use, and medical history. Several measures of physical function and disability will be assessed including the SPPB (Guralnik et al., 1994), Pepper Assessment Tool for Disability (Rejeski et al., 2008), CHAMPS physical activity questionnaire (Stewart et al., 2001), Activities Specific Balance Confidence Scale (Powell and Myers, 1995), FACIT Fatigue Scale (Webster et al., 2003), and Pittsburgh Fatigability Scale (Glynn et al., 2015). Cognitive function will be assessed with the Montreal Cognitive Assessment (Nasreddine et al., 2005), NIH Toolbox cognitive/executive function tests (Weintraub et al., 2013), and an n-back spatial working memory test.

### Walking Assessments

#### Uneven Terrain Walking

**FIGURE 2:**
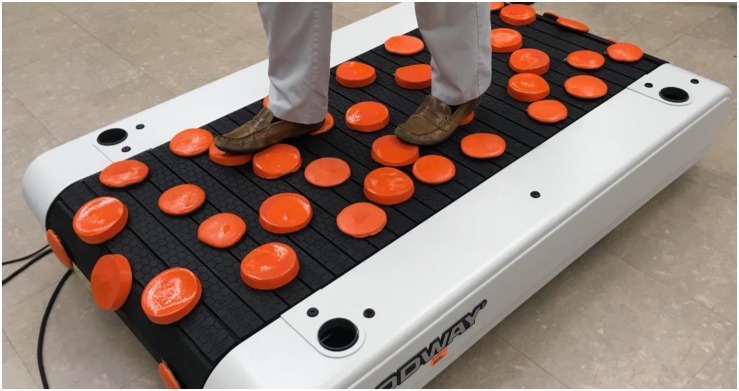
Uneven terrain treadmill surface. The uneven terrain treadmill task involves stepping partially on “disks” that are attached to the treadmill belt. The moderate terrain level is shown here.

Participants will walk on a treadmill with four levels of terrain unevenness, including a flat surface and three uneven terrains of increasing height and variability. The uneven terrain consists of hard foam “disks” (non-compressible) secured to the treadmill belt (Figure 2). For the flat terrain condition all disks will be removed. For the easiest level of terrain all disks will be 1.3 cm in height. For the moderate terrain level there will be a random distribution of two different disk heights, with 50% of the disks at 1.3 cm and 50% at 2.5 cm. The most challenging level of terrain will have a random distribution of three different disk heights, with 50% of the disks at 3.8 cm, 30% at 2.5 cm, and 20% at 1.3 cm. Each terrain will be a separate trial of walking, with the unevenness conditions ordered randomly. The color of the disks will differ for each level of terrain in order to facilitate vivid recollection of the task during subsequent imagined walking for the harmonization aim of the study. Over-ground walking on each terrain will also be performed so that we can measure preferred and maximal walking speed as a secondary performance measure. Each participant will walk at a constant treadmill speed for all terrains to avoid the confounding effect of walking speed on task complexity. The speed will be determined as a percentage of preferred over-ground walking speed (e.g., 75% of over-ground) to compensate for less experience using a treadmill and to ensure participants are capable of walking at a fixed speed across all levels of uneven terrain. Participants will wear a safety harness that is secured to an overhead beam. This harness will stop a fall but does not impede natural walking movements.

#### Complex Walking Tasks

We will conduct a battery of over-ground complex walking tasks including obstacle crossing, dual-task walking, gait initiation, gait speed transitions, and gait termination. During each over-ground walking task, three-dimensional movements of the limbs and body will be measured with a commercially available motion capture system. Likewise, ground reaction force data will be measured by force plates embedded in the floor of the laboratory. For a subset of participants, neuromuscular activation of leg muscles will be measured with electromyography and prefrontal cortical activation will be measured with fNIRS during walking.

#### Community Mobility

Community mobility will be assessed for up to 10 days using a combination of accelerometry and smartwatch global positioning system (GPS) monitoring. Participants will wear a tri-axial accelerometer on the hip. The number of steps per day will serve as the primary outcome because we previously showed it is a strong predictor of MMD (Kheirkhahan et al., 2016). We will examine the number and duration of physical activity bouts at light, moderate, and vigorous activity levels. Participants will also wear a smartwatch with a custom-designed app that captures GPS data and transfers that data to a web-based data visualization program (Kheirkhahan et al., 2019). Data will be processed for excursion size, defined as the maximum distance for each excursion away from home. Average excursion span will also be calculated by averaging the daily maximum distance between all recorded locations away from home.

#### Mobility Function Tests

The 400 m walk test will be used to test for MMD, which is defined as the inability to complete the 400 m walk within 15 min without sitting or receiving help from another person or use of a walker (Hardy et al., 2011; Pahor et al., 2014). Ability to complete the 400 m walk within these criteria is required for enrollment in the study. At each follow up visit, the test will be repeated to assess for emergence of MMD and to categorize participants for the nested case-control sub-study. The SPPB (Guralnik et al., 1994) will also be used to evaluate mobility function. The SPPB is a widely used and validated assessment that includes three sub-components: time to complete an 8-foot walk, time to complete five repeated chair stands, and ability to maintain standing balance with feet together and in semi- and full-tandem foot positions.

#### Imagined Walking

Imagined walking elicits brain activity that is similar to real walking and can reveal information about the neural substrates of locomotion (Hamacher et al., 2015; Stolbkov et al., 2019). Each level of uneven terrain walking will be imagined in a separate trial. To facilitate vivid recollection of the walking tasks, different colored disks will be used for each level of terrain. For EEG and fNIRS, the imagined walking trials will occur within the same sessions as the actual walking trials. For fMRI, participants will be shown standardized images of the appropriately colored uneven terrain surface in motion from a first person perspective. Each individual’s self-reported vividness of motor imagery will be assessed with a questionnaire (Marks, 1995).

### Neuroimaging Procedures and Rationale

#### Electroencephalography (EEG)

**FIGURE 3:**
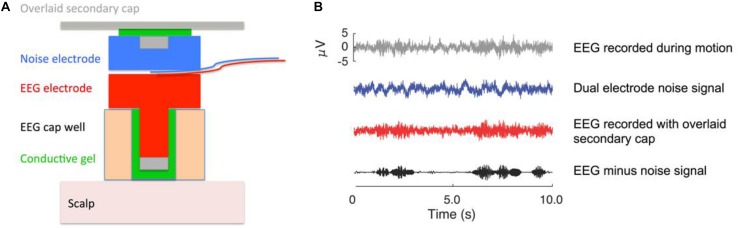
EEG dual electrode design for noise cancellation. **(A)** The dual electrode pair consists of an electrode that records normal EEG and an inverted, noise electrode rigidly coupled to the normal electrode. The noise electrode only records motion artifact and background electrical noise without biological signals. **(B)** Example of EEG data that were recorded on a phantom head (Oliveira et al., 2016a). The gray signal shows data from a normal EEG electrode; the blue signal is the noise recording; the red signal is the scalp recording. The black signal is the isolated neural signal (red minus blue) after noise correction that is used for analysis. The noise subtraction can either occur in the frequency domain for each pair of dual electrodes, or all the electrode signals can be entered into the independent component analysis to filter out the noise content (Nordin et al., 2018, 2019). This figure was created by Dr. Andrew D. Nordin.

The Mind in Motion study will use high-density EEG to quantify electrical brain dynamics during walking. By combining novel dual-electrodes for noise cancellation (Nordin et al., 2018) with Independent Component Analysis and source localization via person-specific inverse electrical head models, it is possible to identify areas of electrical brain spectral power fluctuations with high spatial and temporal precision (Nordin et al., 2019). With this approach, we will determine whether electrical brain activity during actual walking exhibits CRUNCH patterns as participants walk over increasingly uneven terrains. Traditionally, motion artifacts have prevented researchers from using EEG to study human brain function during locomotion (Castermans et al., 2014; Kline et al., 2015). We counter this problem using a custom-built dual EEG electrode (Figure 3) and advanced signal processing, which we have vigorously validated (Oliveira et al., 2016a, b; Oliveira et al., 2017a, b; Nordin et al., 2018, 2019). After fitting a 128-channel head cap, we will record the location of each electrode relative to bony landmarks with a digitization pen. The dual-electrodes are inserted into the cap, mounted back-to-back such that a primary electrode faces the scalp while the secondary electrode faces away from the scalp. A second cap of conductive material is placed over the electrodes, which comes in direct contact with the recording surface of the secondary electrodes. The conductive cap serves as artificial skin so that these inverted electrodes are not completely electrically isolated from each other during data collection. With this approach, each secondary electrode captures electrical noise and movement artifact only, which can then be subtracted from the recordings of the primary electrode to better distinguish physiological components of the EEG signal (Nordin et al., 2018, 2019).

We will apply an adaptive mixture independent component analysis algorithm (AMICA) that generalizes infomax (Bell and Sejnowski, 1995; Lee et al., 1999) and multiple mixture ICA approaches, to parse EEG signals into spatially static, maximally independent component processes. Each participant will have an anatomical Magnetic Resonance Imaging (MRI). Using the T1-weighted whole-head magnetic resonance image of each participant, we will create a four-layer, ∼20,000-node boundary element method (BEM) model representing the brain, cerebrospinal fluid, skull, and scalp. The digitized electrode locations will be transferred to each participant-specific head model. The independent components will be classified as electrocortical sources or muscle sources based on inspection of their power spectra and the locations of their equivalent current dipoles (Onton and Makeig, 2006, 2009; Onton et al., 2006). Electrocortical sources will be clustered across participants using *k*-means clustering on vectors jointly coding differences in equivalent dipole locations and power spectra (Gwin et al., 2010, 2011).

#### Functional Near Infrared Spectroscopy (fNIRS)

Functional near infrared spectroscopy, a neuroimaging technique for assessing cortical activity, will be recorded during actual and imagined walking over the varying levels of terrain unevenness. Specifically, task-dependent changes in oxygenated and deoxygenated hemoglobin concentration are used to infer cortical activity based on the known coupling between neuronal activity and hemodynamic response (neurovascular coupling) (Scholkmann et al., 2014). fNIRS will also be used to gauge cortical activity during the over-ground complex walking tasks. Prefrontal cortical activity will be recorded with a commercially available multi-channel fNIRS device. Cortical activation under each optode pair will be estimated as the change in oxygenated hemoglobin concentration (ΔO2Hb) between the walking and resting baseline conditions (Perrey, 2014). fNIRS data will be compared to EEG and fMRI data for the harmonization aim, allowing us to determine common features of mobility-related brain activity across different functional imaging modalities.

#### Magnetic Resonance Imaging (MRI)

Several MRI approaches will be used in this study to measure CRUNCH-related factors affecting control of walking. Functional magnetic resonance imaging (fMRI) will be recorded during imagined walking over the varying levels of uneven terrain. These data will be compared to EEG and fNIRS data during actual and imagined walking as part of the harmonization aim of the study. We will also acquire several measures of brain structure and function to determine whether they may be underlying mechanisms of CRUNCH indices. High resolution T1 scans will be used to measure brain volume, regional gray matter volumes, and cortical thickness as well as for spatial normalization of fMRI and EEG data. Resting state functional connectivity MRI (fcMRI) will measure network segregation. Diffusion weighted MRI (dMRI) will assess structural integrity of white matter tracts. Pseudo continuous arterial spin labeling (PCASL) MRI will measure cerebral blood flow.

#### Sensory Function

Sensory function will be evaluated with clinical assessments of tactile somatosensation, vision, vestibular function, and pain to determine whether they are underlying correlates of CRUNCH scores and walking ability. Tactile somatosensation on the sole of the foot will be measured with two-point discrimination and Semmes-Weinstein monofilaments. Vision will be tested with a standard Snellen Eye Chart, as well as with the Useful Field of View Test which measures the visual area over which information can be extracted at a brief glance (Ball et al., 2002). Vestibular function will be assessed using a timed Romberg test, which is a test of static standing postural stability with eyes open versus eyes closed (Lanska and Goetz, 2000). Multiple dimensions of acute and chronic pain will be assessed. Participants will be interviewed about the frequency, intensity, and location of pain in each region of the body. They will also be asked to rate any pain that they feel during the 400 m walk test (i.e., movement evoked pain) on a scale of 0–100 (Cruz-Almeida et al., 2017). A pressure pain threshold will also be measured by applying standardized pressure to the anterior thigh until the participant begins to perceive it as being painful.

### Data Reduction, Statistical Analyses, and Power Analysis

For all neuroimaging modalities (EEG, fNIRS, fMRI), brain regions exhibiting CRUNCH responses will initially be identified as those that increase their activity with increasingly uneven terrain conditions for actual and/or imagined walking. For EEG, we expect increased theta spectral power in frontal brain regions (prefrontal and anterior cingulate). For fNIRS, we expect increased oxygenated hemoglobin concentrations in the prefrontal cortex. For fMRI, we expect increased BOLD responses in frontal brain regions (prefrontal and anterior cingulate). The primary CRUNCH-related outcome measure will be the interpolated *x*-axis (terrain unevenness) level at which brain activity exhibits an inflection point (plateau or decline in brain activity). This “CRUNCH score” is affected by both over-recruitment of brain activity and resource recruitment ceiling. The CRUNCH score will be tested for association with walking function in cross-sectional and longitudinal analyses. This score will also be compared across neuroimaging modalities for the harmonization aim.

We will use multiple linear regression for cross-sectional analysis and generalized linear mixed models for longitudinal continuous data to assess CRUNCH and the mobility outcomes. For MMD, we will use survival analyses such as Cox proportional hazards regression to evaluate CRUNCH and risk of MMD. Failure time is measured from the date of first 400 m walk visit to the visit date when failure is recorded. Censoring is defined as the last date of contact known to be free of MMD. For the nested case-control sub-study, conditional logistic regression will assess the relation between case status and change in CRUNCH during walking. Change will be defined as an absolute difference between follow-up and baseline values.

Power analyses considered both cross-sectional and longitudinal associations. Regarding the former, the proposed sample of 220 (200 older adults and 20 young adults) gives 80% power to detect a correlation as small as 0.19 when the null correlation is zero (at 2-sided 0.05 significance level). Some measurements (e.g., dMRI, PCASL MRI) are being collected on a pseudo-random sample of 90 participants, which is capable of detecting a univariate correlation between CRUNCH measures and proposed mechanisms as small as 0.29 when the null correlation is zero (at 2-sided 0.05 significance level). For longitudinal analyses, we will follow older adults (*n* = 200) for 1.25–3.5 years and we conservatively estimate a 5% loss of follow-up information resulting in an effective sample size of 190. This effective sample has 80% power to detect 0.2 standard deviations in the change in walk speed per one standard deviation difference in the CRUNCH primary predictor. For assessing mechanisms contributing to MMD, there is 80% power (0.05 two-sided alpha) to detect a hazard ratio of 1.45 per one standardized difference in a predictor of interest with a projected 30% MMD event rate (as observed in LIFE and LIFE-pilot studies) (Investigators et al., 2006; Pahor et al., 2014). For powering the case-control comparison, we expect a larger change in CRUNCH in cases versus controls. We anticipate a sample of 45 cases that will be matched with 45 controls (90 participants total, which will give 80% power to detect a 0.6 standard deviation change (or difference) in the CRUNCH predictor.

## Discussion

### The CRUNCH Framework Applied to Motor Control

The CRUNCH framework has emerged from two decades of cognitive neuroimaging research in older adults (Reuter-Lorenz and Cappell, 2008; Schneider-Garces et al., 2010). Although the framework has largely been developed and tested in the context of cognitive function, we believe based on our pilot data that it is also highly relevant for complex motor functions including walking. Our preliminary findings from an fMRI study on finger sequencing, in which older adults performed sequences of six button presses, support this notion (Cooke et al., 2016). The task had seven levels of task complexity, with each level recruiting the use of additional fingers and/or increasing the number of transitions between fingers during the sequence. Regions following the CRUNCH framework included the cerebellum and frontoparietal cortices, which were recruited in a compensatory manner for low difficulty levels and then reached a maximum capacity at higher difficulty levels. In the context of walking, we have previously shown that EEG spectral power fluctuations differ according to the complexity of the walking conditions (Sipp et al., 2013; Kline et al., 2015, 2016; Bradford et al., 2016; Oliveira et al., 2016a, b). For example, dorsolateral prefrontal cortex shows increased theta spectral power when young healthy participants walk on a treadmill-mounted balance beam compared to walking on the flat treadmill belt (Sipp et al., 2013). fNIRS has also provided evidence of “CRUNCH-like” changes in cortical activity during walking. Evidence of prefrontal over-recruitment during typical steady state walking has been reported in several populations with compromised mobility, including older adults (Chen et al., 2017; Mirelman et al., 2017; Hawkins et al., 2018) and people with stroke (Hawkins et al., 2018; Chatterjee et al., 2019), Parkinson’s disease (Maidan et al., 2016a,b), and multiple sclerosis (Hernandez et al., 2016). This is potentially consistent with recruitment of attentional resources to maintain functional walking and compensate for weakness, sensory loss, and/or other impairments affecting control of walking (Clark, 2015). Some studies have also provided insight to the availability of resource reserves beyond what is used during typical walking. One of these prior studies measured prefrontal activity with fNIRS in older and young adult participants while performing typical and dual-task walking (with verbal phonemic fluency) (Hawkins et al., 2018). Prefrontal activity was lower in younger individuals compared to older during typical walking. Furthermore, prefrontal activity in young adults was much lower for typical walking versus dual-task walking, suggesting high reserves of brain recruitment resources during typical walking. In contrast, the difference in prefrontal recruitment between tasks was modest in older adults. These data are consistent with over-recruitment of prefrontal resources in older adults during typical steady state walking. Another study reported data suggesting a ceiling effect for prefrontal recruitment during complex walking in adults post-stroke (Chatterjee et al., 2019). The participants performed dual-task walking (with serial-7 subtraction) while prefrontal activity was recorded with fNIRS. Across participants, lower prefrontal activity during this task was associated with poorer global cognitive function (Mini-Mental State Exam). This finding suggests that cognitive deficits may lower the ceiling of available brain resources that can be recruited during a complex walking task. Other studies of obstacle walking have reported that people who exhibit smaller increases in prefrontal recruitment during obstacles relative to typical walking (possibly an indicator of inadequate reserves) also have a steeper decline in walking speed between tasks (Clark et al., 2014b; Hawkins et al., 2018).

In the Mind in Motion study, we will build on the aforementioned evidence to understand how mobility function in older adults is linked to CRUNCH indices of brain over-recruitment and ceiling effects. We will also investigate several age-related central and peripheral neural markers that may contribute to CRUNCH effects. Central mechanisms will be investigated with magnetic resonance imaging (MRI). For example, the well-known age-related loss of gray matter volume and cortical thickness (Rosano et al., 2007; Lemaitre et al., 2012) will be investigated for links to a possible reduction in the brain activity resource ceiling, thus contributing to CRUNCH. We will also assess resting state fcMRI to test whether lack of segregation between cerebral networks is associated with over-recruitment at low levels of task complexity and reduced resource ceiling. Recent work has demonstrated that individual networks become less segregated with age (Antonenko and Floel, 2014; Chan et al., 2014), and that decreasing segregation is associated with poorer motor function (King et al., 2017). Likewise we will use dMRI to test whether regional measures of white matter microstructure are associated with a lower resource ceiling. We have previously shown that individual differences in white matter microstructure of the cingulum bundle are correlated with maximum grip strength in healthy older adults (Hirsiger et al., 2016), and corpus callosum white matter microstructure is associated with complex bimanual coordination in healthy older adults (Fling and Seidler, 2012). Further, older adults with altered white matter microstructure exhibit *more* functional brain activity in surrounding regions, suggesting that over recruitment in older adults may occur at least in part as compensation for white matter declines (Daselaar et al., 2015). Finally, pCASL MRI will be used to measure cerebral blood flow (Dai et al., 2008). Cerebral blood flow declines with normal aging in many brain regions (Bangen et al., 2009) and is associated with cognitive performance (Chapman et al., 2013). Furthermore, reduced blood flow velocity (measured with transcranial Doppler ultrasound) is associated with slower gait speed in older adults (Ezzati et al., 2017). Also of interest is the finding that some brain regions exhibit greater cerebral blood flow in older age, particularly for individuals with white matter impairment, suggesting a compensatory response (Kraut et al., 2008).

Peripheral measures will be used to assess the effects of sensory function on CRUNCH indices of brain activity. These measures will include clinical assessments of tactile somatosensation, pain, vision, and vestibular function. Impaired somatosensory function (tactile, pain) in older adults is associated with deficient balance and slower walking speed (Cruz-Almeida et al., 2014, 2017). Our own recently acquired data also show an association between impaired tactile sensation on the sole of the foot (Semmes-Weinstein monofilament testing) and higher recruitment of prefrontal cortex measured with fNIRS during typical steady state walking (Clark unpublished findings). When tactile sensory feedback in older adults is augmented by wearing textured shoe insoles, we have observed a reduction in prefrontal activation as measured by fNIRS during walking in older adults (Clark et al., 2014a). Cumulatively these findings suggest that tactile sensory impairment in the feet may contribute to less automatic control of walking (Clark, 2015) and CRUNCH compensatory over-recruitment of attentional resources. We have further shown that visual restriction imposed during walking is associated with greater sensorimotor cortical EEG power spectral fluctuations during walking (Oliveira et al., 2017b).

### Selection of Uneven Terrain as the Primary Walking Task

The task that we selected for the Mind in Motion study is walking on flat and progressively more uneven terrain. Other tasks were considered during the development stage of the study, but we came to the consensus that uneven terrain walking best met the following key criteria. First, the task should have a relatively high degree of ecological validity. Various levels of uneven terrain are present in the natural environment, more so than for other complex walking tasks that have been reported in the literature like split belt treadmill paradigms or slip perturbations. Terrain has been identified as one of several domains of walking that is crucial to successful community ambulation (Patla and Shumway-Cook, 1999). The majority of falls in community-dwelling older adults occur outside the home and a large percentage of those falls involve uneven surfaces (Li et al., 2006). Prior evidence shows that walking on uneven terrain requires real-time sensorimotor adjustments that are non-uniform and unpredictable. Even for healthy adults, uneven terrain walking elicits modification of spatiotemporal gait parameters and limb kinematics to meet the demands of the task (MacLellan and Patla, 2006; Wade et al., 2010; Voloshina et al., 2013). For example, in our prior work comparing a low level of uneven terrain to flat walking in healthy young adults, participants had 4% decreased step length, 22% increased step length variability, 36% increased step width variability, 28% increase in positive/concentric work at the knee, and 62% at the hip, 26% increased negative/eccentric work at the knee, and altered neuromuscular activation and co-activation across seven leg muscles (Voloshina et al., 2013). These findings demonstrate that small differences in terrain can create a complex walking environment with considerable modifications to gait biomechanics and neural control.

Second, the task should primarily challenge sensorimotor aspects of brain control of walking. Uneven terrain meets this criterion by altering the somatosensory feedback from the walking surface on a step by step basis, and by requiring the participant to voluntarily adapt foot placement (altering step length and mediolateral foot placement) to ensure that the foot is positioned on the uneven terrain in a manner that contributes to stable support and propulsion of the body throughout stance phase. Our focus on sensorimotor control differentiates our study from the more common dual-task paradigms that have been used previously to probe brain control of walking. Dual-task paradigms typically combine walking with a separate cognitive or motor task, such that control resources must be divided. In the case of walking with a cognitive task, the control resources are being diverted toward an overtly cognitive task as opposed to challenging motor control. In contrast, walking on uneven terrain requires a heightened and focused allocation of control resources to the task of walking.

A third criterion is that the walking task must allow for objective incremental changes in task complexity, consistent with evaluating CRUNCH-related outcomes. For the uneven terrain task, all participants will be tested on the same four levels of terrain unevenness. The more complex terrain surfaces have both an increased maximal height of disks as well as more variety in disk height. These factors make the walking surface less predictable as complexity increases, which systematically increases the importance of somatosensory feedback and the need to recruit attentional resources in the control of limb movement and posture.

A fourth criterion was to select a walking task that requires stable recruitment of a relatively small number of brain regions over the duration of each walking trial. Within each level of uneven terrain walking, we expect a relatively sustained level of brain resource demand for each step taken by the participant. This differs from some other complex walking tasks, for example obstacle walking where brain resource demand may vary for obstacle crossing steps versus typical steps (i.e., when not crossing over an obstacle). For different levels of uneven terrain walking, we expect that differences in brain activity will primarily be limited to prefrontal, motor, somatosensory, parietal, and cingulate cortices. This is beneficial for the whole-brain EEG analysis, because brain activity across many regions would dilute the statistical power for detecting changes within primary regions.

### Comprehensive Assessment of Walking and Mobility

The Mind in Motion study will thoroughly characterize walking and mobility function in each participant. As with our primary task of uneven terrain, we prioritized ecological validity by including several secondary walking assessments that capture domains important to community mobility including cognitive dual-tasking, obstacle crossing, temporal demands (e.g., fast and slow walking speeds), and postural gait transitions (e.g., initiation and termination) (Patla and Shumway-Cook, 1999; Balasubramanian et al., 2014). Older adults are more likely to show performance deficits on these complex walking tasks than for typical steady state walking (Shumway-Cook et al., 2007), which will improve sensitivity for detecting individual differences and changes in walking function over time. The selected tasks are also widely studied in the literature, which will facilitate comparison of our findings to prior and future work. In a subset of participants and tasks, we will also acquire simultaneous recording of biomechanics, electromyography, and fNIRS to provide a rich data set for investigating relationships between central neural control and resultant neuromuscular activation and movement.

Cognitive dual-task walking performance will contribute valuable insights into cognitive-motor interactions. Prior studies support that impaired walking performance during the simultaneous execution of cognitive tasks predicts and/or is associated with increased mobility disability (Verghese et al., 2002, 2012; Auvinet et al., 2017). Obstacle crossing is challenging for older adults because it requires increased use of both cognitive and neuromuscular effort (Patla and Shumway-Cook, 1999). The inability to negotiate such environmental hazards is among the greatest contributor to falls in older adults (Al Faisal et al., 2016). We will use spatiotemporal measures of crossing behavior and measures of dynamic stability to investigate participants’ ability to perform an obstacle crossing task. Gait initiation is a challenging neural control task because it is a volitional transition from a condition of a static stable support to a continuously unstable posture during walking (Hass et al., 2005; Nocera et al., 2010; Deshpande et al., 2013; Lee et al., 2016). Because of this, gait initiation is used as an investigative tool to provide insight into postural-gait control and the changes that occur with advancing age and disability (Jian et al., 1993; Halliday et al., 1998; Polcyn et al., 1998; Chang and Krebs, 1999; Martin et al., 2002; Amano et al., 2015; Skinner et al., 2015). Center of pressure (COP) adjustments and the magnitude of separation between the COP and center of mass during gait initiation are sensitive indicators of increased fall risk and mobility disability (Chang and Krebs, 1999; Sparrow and Tirosh, 2005). Gait termination is the transition from steady state walking to quiet stance and requires the effective arrest of momentum. Both predictive and reactive gait termination are important to everyday walking and are sensitive markers of disability and fall risk (Wikstrom et al., 2010; Wikstrom and Hass, 2012). The number of steps required to terminate gait, as well as the corresponding ground reaction forces, provide insights into task performance.

In addition to lab-based assessments, the Mind in Motion study will also investigate “real world” community mobility using actigraphy and GPS smartwatch technology. Understanding walking behavior in the natural environment, and how laboratory derived brain and biomechanical analyses predict these abilities, is of great importance. Primary outcomes will include the number of steps per day and distance (excursion) traveled away from home, where smaller values indicate more compact traveling. These data will create a detailed picture of community mobility (walking, driving, travel, etc.) and is akin to the “life-space” concept where the distance of movement away from home shrinks with aging and mobility loss (Stalvey et al., 1999).

We will also conduct well-established, standardized, clinically feasible tests of mobility function including the 400 m walk test and the SPPB. Ability to complete the 400 m walk test will be used to define the “MMD” outcome, and is an objective, reliable (Rolland et al., 2004) and well-validated approach. Average walking speed from the 400 m walk will also be assessed as a performance outcome measure. Walking speed is strongly and consistently associated with a variety of poor functional outcomes and mortality in older adults (Guralnik et al., 2000; Newman et al., 2006; Studenski et al., 2011).

### Harmonizing Data Across Neuroimaging Modalities

Harmonizing neuroimaging data refers to understanding the consistency of findings across EEG, fMRI, and fNIRS. We seek to determine which CRUNCH-related hallmarks of brain activity are shared or independent across neuroimaging modalities, which will facilitate more widespread application of the study findings. For instance, this information will be useful for researchers who are planning new studies using their single imaging modality of choice. It may also be useful for re-interpreting prior data in the literature for CRUNCH-related findings that were not previously evident. Data will be compared across EEG and fNIRS for actual walking on uneven terrain. All three neuroimaging modalities (including fMRI) will be compared for imagined walking. Prior studies have reported differences in brain activity for real versus imagined walking, such as greater primary sensorimotor cortical activity in real walking and greater basal ganglia activity in imagined walking (Stolbkov et al., 2019). Other regions of importance exhibit similar activity such as prefrontal, supplementary motor, visual, and cerebellar regions. We anticipate substantial overlapping activity across modalities, and seek to assess whether these regions exhibit similar CRUNCH-related responses during uneven terrain walking. Raw data from each modality will be resampled to a common temporal resolution, and transfer functions will be used to model predicted versus actual activation in brain regions of interest. Qualitative spatial overlap of brain activity across modalities will also be used to understand which brain regions/networks are responsive to variations in terrain unevenness and/or correlated with walking performance.

### Future Impact

The Mind in Motion study will establish hallmarks of normal and abnormal brain recruitment during walking as well as identify key intervention targets. Measuring CRUNCH-related indices of brain activity is significant because these indices reflect potentially modifiable targets for intervention. Furthermore, these indices may serve as identifiable risk factors that could warrant earlier interventions to mitigate declines in mobility function. The results will inevitably lead to targets ideal for fitting particular interventions to an individual (i.e., personalized medicine). As an example, two important CRUNCH indices to target are over-recruitment of brain resources and having a low ceiling of available brain resources to cope with task demand. Interventions designed to retrain efficient brain activity through adaptive physical rehabilitation (i.e., motor learning) are a logical choice. For example, increasingly complex walking conditions can be practiced and mastered gradually to promote re-learning of a more automatic movement control strategy that is less demanding of brain resources. Over-recruitment might also be addressed by augmenting sensory inputs to the central nervous system. For example, a prior study has reported reduced prefrontal activity during walking when wearing textured shoe insoles (Clark et al., 2014a). A similar benefit might be attainable by addressing other sensory issues such as visual impairment, movement-evoked pain, or proprioception impairments. Other options may include physical exercise (Fernandes et al., 2017), pharmacological interventions, non-invasive neuromodulation [e.g., direct current stimulation (Woods et al., 2016)], and combinations of these approaches. Results from the Mind in Motion study will provide an important foundation for developing, testing, and interpreting these therapeutic interventions.

### Alternative Strategies

It is possible that we will not observe the expected CRUNCH-like pattern of increased brain activity with greater task complexity. For example, brain activity may not reach a ceiling due to insufficient task challenge or there might be a less nuanced “all or nothing” recruitment of key brain regions that warrants collapsing the data across two or more levels of terrain complexity. Given the existing strong evidence for CRUNCH during cognitive tasks in older adults, it will be important to learn whether this model translates well to walking function. If necessary, we are prepared to consider other neural control frameworks for interpreting our walking data. It is also possible that the brain regions recruited during uneven terrain walking may differ from what we expect. This will also be an important finding, as a better understanding of which brain networks relate to complex walking performance can still lead to new intervention targets and strategies.

## Author Contributions

DC, TM, DF, CH, and RS provided major contributions to manuscript writing and study design. BB, YC-A, PR-L, and MP provided secondary contributions. All authors approved the content of this manuscript.

## Conflict of Interest

The authors declare that the research was conducted in the absence of any commercial or financial relationships that could be construed as a potential conflict of interest.
