# Corrigendum: Multimodal Imaging of Brain Activity to Investigate Walking and Mobility Decline in Older Adults (Mind in Motion Study): Hypothesis, Theory, and Methods

**DOI:** 10.3389/fnagi.2020.00063

**Published:** 2020-03-04

**Authors:** David J. Clark, Todd M. Manini, Daniel P. Ferris, Chris J. Hass, Babette A. Brumback, Yenisel Cruz-Almeida, Marco Pahor, Patricia A. Reuter-Lorenz, Rachael D. Seidler

**Affiliations:** ^1^Department of Aging and Geriatric Research, University of Florida, Gainesville, FL, United States; ^2^Brain Rehabilitation Research Center, Malcom Randall VA Medical Center, Gainesville, FL, United States; ^3^Department of Biomedical Engineering, University of Florida, Gainesville, FL, United States; ^4^Department of Applied Physiology and Kinesiology, University of Florida, Gainesville, FL, United States; ^5^Department of Biostatistics, University of Florida, Gainesville, FL, United States; ^6^Pain Research and Intervention Center of Excellence, University of Florida, Gainesville, FL, United States; ^7^Department of Psychology, University of Michigan, Ann Arbor, MI, United States

**Keywords:** mobility, walking, older adults, brain, neuroimaging, EEG, MRI, fNIRS

In the original article, there was a mistake in the legend for **Figure 3** as published. The legend failed to acknowledge the contribution of Dr. Andrew D. Nordin in creating this figure. The correct legend appears below.

**Figure 3**. EEG dual electrode design for noise cancellation. **(A)** The dual electrode pair consists of an electrode that records normal EEG and an inverted, noise electrode rigidly coupled to the normal electrode. The noise electrode only records motion artifact and background electrical noise without biological signals. **(B)** Example of EEG data that were recorded on a phantom head (Oliveira et al., 2016a). The gray signal shows data from a normal EEG electrode; the blue signal is the noise recording; the red signal is the scalp recording. The black signal is the isolated neural signal (red minus blue) after noise correction that is used for analysis. The noise subtraction can either occur in the frequency domain for each pair of dual electrodes, or all the electrode signals can be entered into the independent component analysis to filter out the noise content (Nordin et al., 2018, 2019). This figure was created by Dr. Andrew D. Nordin.

The authors apologize for this error and state that this does not change the scientific conclusions of the article in any way. The original article has been updated.
